# A novel *Streptococcus pneumoniae* human challenge model demonstrates Treg lymphocyte recruitment to the infection site

**DOI:** 10.1038/s41598-022-07914-w

**Published:** 2022-03-07

**Authors:** Gabriella Szylar, Riccardo Wysoczanski, Helina Marshall, Daniel J. B. Marks, Ricardo José, Michael R. Ehrenstein, Jeremy S. Brown

**Affiliations:** 1 Centre for Inflammation and Tissue Repair, UCL Respiratory, Division of Medicine, Rayne Building, 5 University Street, London, WC1E 6JF UK; 2 Centre for Rheumatology, UCL Division of Medicine, Rayne Building, 5 University Street, London, WC1E 6JF UK; 3 Centre for Molecular Medicine, UCL Division of Medicine, Rayne Institute, 5 University Street, London, WC1E 6JF UK

**Keywords:** Immunology, Microbiology, Diseases, Medical research

## Abstract

To investigate local tissue responses to infection we have developed a human model of killed *Streptococcus pneumoniae* challenge by intradermal injection into the forearm. *S. pneumoniae* intradermal challenge caused an initial local influx of granulocytes and increases in TNF, IL6 and CXCL8. However, by 48 h lymphocytes were the dominant cell population, mainly consisting of CD4 and CD8 T cells. Increases in local levels of IL17 and IL22 and the high proportion of CD4 cells that were CCR6^+^ suggested a significant Th17 response. Furthermore, at 48 h the CD4 population contained a surprisingly high proportion of likely memory Treg cells (CCR6 positive and CD45RA negative CD4^+^CD25^high^CD127^low^ cells) at 39%. These results demonstrate that the intradermal challenge model can provide novel insights into the human response to *S. pneumoniae* and that Tregs form a substantial contribution of the normal human lymphocyte response to infection with this important pathogen*.*

## Introduction

Despite the widespread use of antibiotics and vaccination bacterial pathogens remain an important cause of mortality and morbidity globally^1,2^. Bacterial infection will cause a local and sometimes a systemic inflammatory response, recruitment to the site of infection of both antigen-specific and other white cell populations, and systemic effects on innate and humoral soluble immune effectors, including antibody. A detailed understanding of these pathogen/host interactions is essential to define how individual pathogens establish serious infections and why certain subjects are at higher risk. However, the available methodologies for investigating host pathogen interactions have significant weaknesses. For example, the effects of anatomy, cell to cell interactions, and the large range of different structural and immune cells involved in bacterial infection cannot be accurately replicated using in vitro experiments with human cells, and data obtained from animal models may not reflect bacterial interactions with human cells^3^. Furthermore, animal models usually do not replicate the complex immune background found in humans, who may have adaptive immune responses to the pathogen under investigation due to previous exposure (often on multiple occasions), and in whom co-morbidities or the effects of age can substantially alter immune responses.

Human infection challenge models have been used to overcome some of these limitations of cell and animal infection models of infection. For example, a nasopharyngeal human colonisation model has been established for the important Gram positive pathogen *Streptococcus pneumoniae*, a common nasopharyngeal commensal species that is also a common cause of severe bacterial infections such as pneumonia, septicaemia and meningitis and causes approximately 1.3 million deaths per year of death^4,5^. Live *S. pneumoniae* human infection models have made important novel findings about innate and adaptive immune responses to nasopharyngeal colonisation, as well as bacterial/epithelial interactions^6–10^. In addition, the model can be used to test the efficacy of vaccines^11,12^. However, the human challenge colonisation model only reflects bacterial/host interactions at a mucosal site during colonisation, and there is a need for an additional human model that can be used to characterise the inflammatory and adaptive immune response to systemic *S. pneumoniae* infection, including the effects of different host factors (eg age, immunosuppression) on these responses. The inflammatory and immune response to *S. pneumoniae* have been defined for animal models, which show that *S. pneumoniae* pneumonia initially causes a rapid influx of neutrophils to the sites of infection associated with high levels of pro-inflammatory cytokines and chemokines (eg TNFα and IL-6) followed by recruitment of monocytes and lymphocytes (including Tregs) over 24–48 h^13–19^. However, these data have generally been obtained in immune naïve mice without prior exposure to *S. pneumoniae*, whereas all humans have multiple episodes of prior exposure to *S. pneumoniae* resulting in significant adaptive immune responses that are difficult to accurately replicate in an animal model. These adaptive immune responses to *S. pneumoniae* that are present in all individuals are likely to affect the pattern of the initial inflammatory response, as well as alter the pattern of lymphocyte recruitment due to the presence of antigen specific B cells and T cell subsets^20–26^. Using samples from patients with active *S. pneumoniae* infections to corroborate data obtained using animal models is difficult due to an inability to sample the site of infection (especially at repeated time points) or assess these responses before clinical presentation, and because the effects of treatment alter the natural course of infection.

Overall, there is a strong scientific need for an *S. pneumoniae* human challenge model that can be used to investigate the immune response to systemic infection. Direct infection of human lungs is not a practical model, and infection using live *S. pneumoniae* is also not possible due to the significant risk of severe infection. Hence, as an alternative human challenge model suitable for investigating the inflammatory and immune responses to *S. pneumoniae* we have developed a human model of killed *S. pneumoniae* challenge by intradermal injection into the forearm. This model uses the skin as a window to the human immune response to *S. pneumoniae*, and allows recovery of cell and extracellular fluid at the site of infection and therefore the characterisation of soluble and cellular inflammatory and immune responses to *S. pneumoniae* over time. Here we describe this model, and use the model to define the timing and nature of the healthy adult human CD4 lymphocyte response to *S. pneumoniae* challenge.

## Results

### An intradermal killed* S. pneumoniae* challenge model causes a local inflammatory response

**Figure 1:**
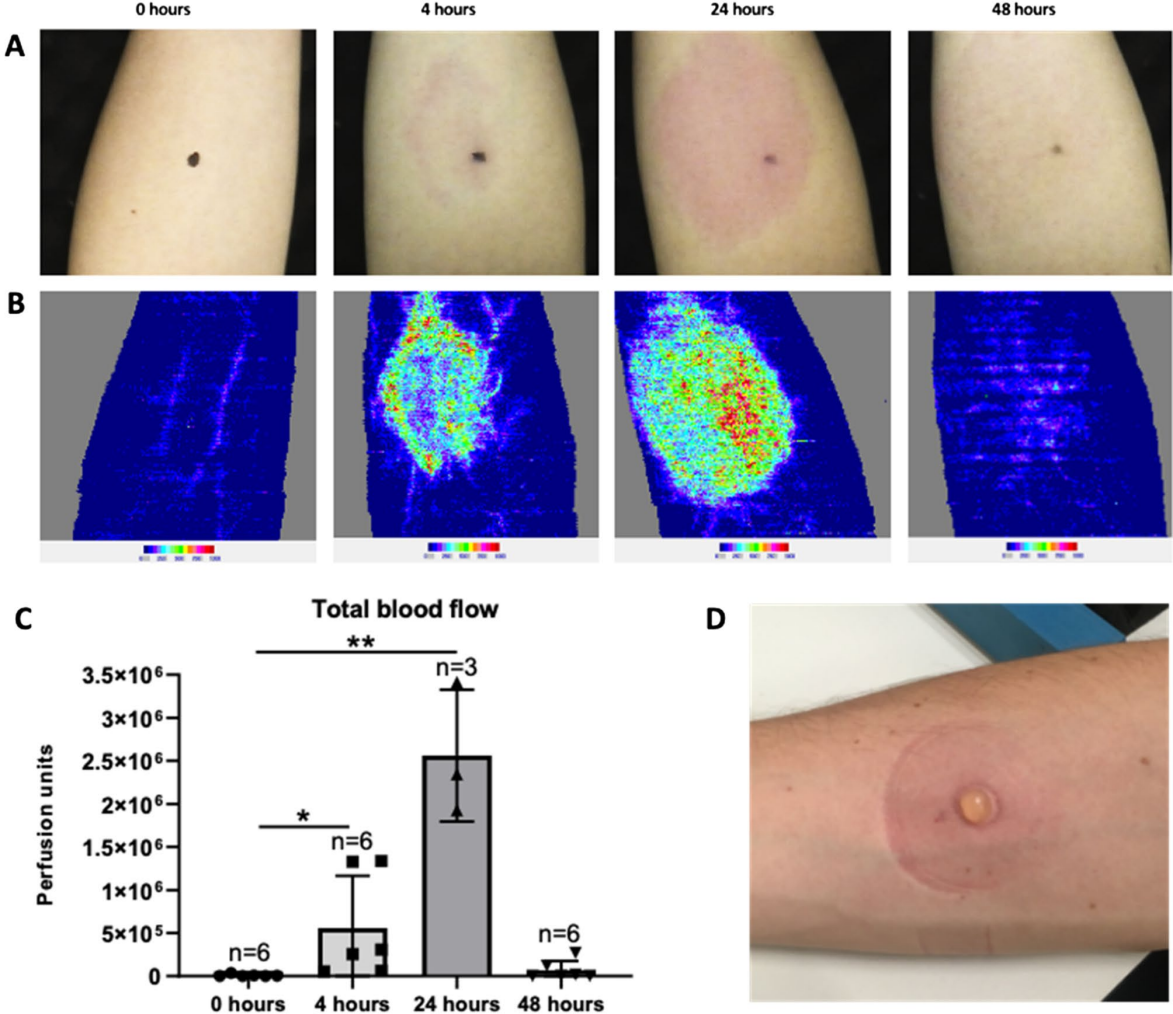
Blood flow to the site of intradermal injection of killed S. *pneumoniae* in the forearms of healthy volunteers. Intradermal inoculation of 7.5 × 10^5^ CFUs of UV-killed TIGR4 S. *pneumoniae* was followed over 48 h by Laser Doppler scans of the injection area. (**A**) Representative photographs and (**B**) Doppler scans images at the specified time points from one volunteer. (**C**) Mean blood flow calculated from Doppler scans of the forearms of 6 volunteers for the 0 h (pre-injection), 6 volunteers at 4 h and 48 h post-injection, and 3 volunteers at the 24 h post-injection time points. Data was analysed using Laser Doppler moorLDI software and presented as arbitrary perfusion units (sum of the pixels above 300 perfusion units and mean blood flow signal). Data presented as means, error bars represent SDs, and statistical analysis was done using Kruskal–Wallis test (*p* < 0.01) with Dunn's multiple comparisons test (*p < 0.05, **p < 0.01). (**D**) Photograph of a blister raised at the site of injection using a negative pressure instrument.

Healthy volunteers were given intradermal injections of 7.5 × 10^5^ UV-killed *S. pneumoniae* TIGR4 strain suspended in 100 μl of saline into each forearm. A variable sized patch of erythema developed at the site of infection, fading by 48 h (Fig. 1A). There were no systemic side effects, skin breakdown, or persisting skin changes. Blood flow at the injection site at the injection site was measured using Laser Doppler scanning (Fig. 1B), and was increased at 4 h post-injection before peaking at 24 h (Fig. 1B,C). By 48 h post-challenge, blood flow and visible inflammation had returned to levels close to baseline. These data confirm intradermal injection of killed *S. pneumoniae* caused an inflammatory response that was detectable by 4 h post-challenge, peaked at approximately 24 h, and was resolving by 48 h.

### Cell recruitment in response to intradermal *S. pneumoniae* injection

**Figure 2:**
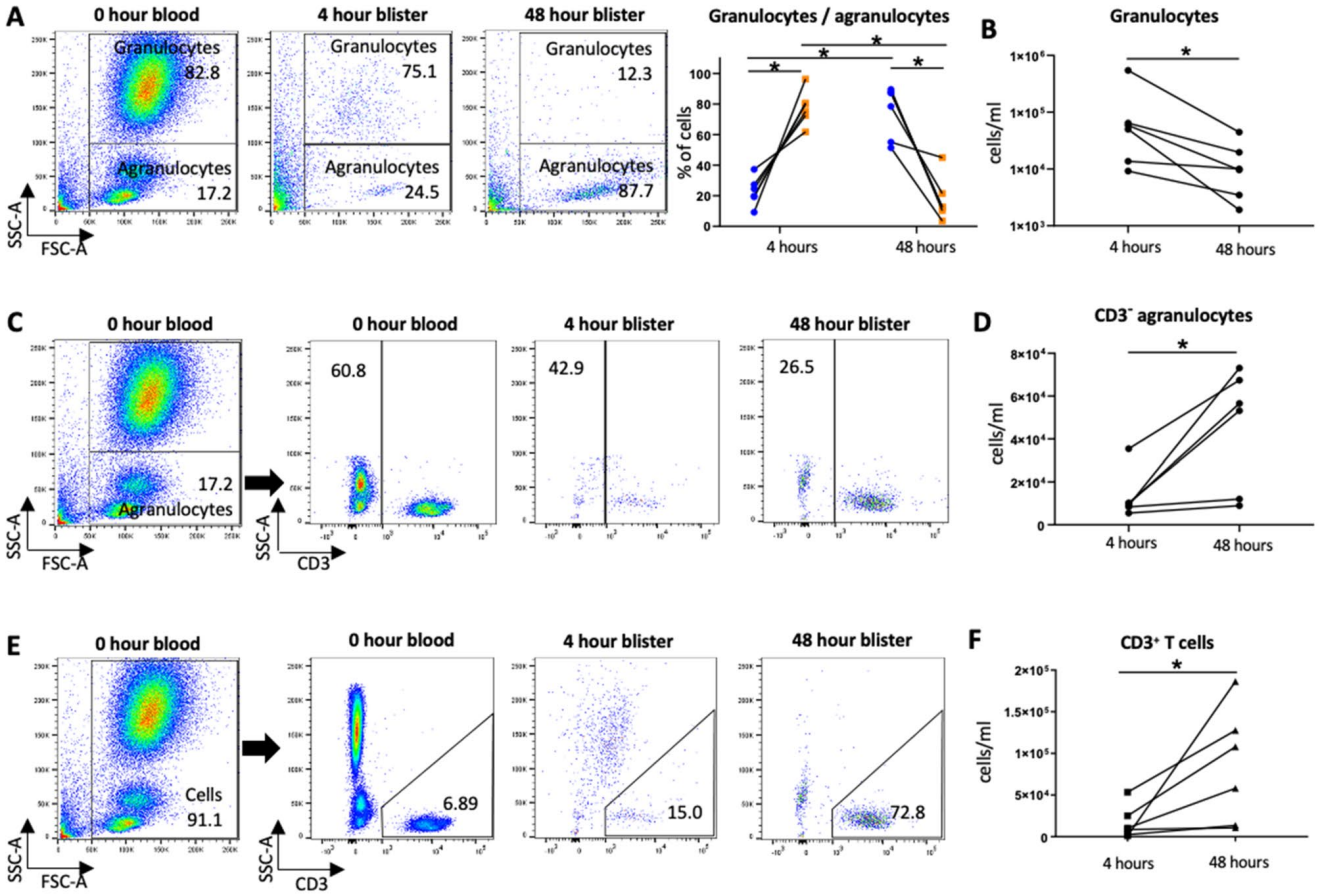
Cell population 4 and 48 h post-intradermal injection of killed *S. pnuemoniae* in the forearms of healthy volunteers. Data were obtained by flow cytometry of blister fluid obtained 4 and 48 h after intradermal inoculation of 7.5 × 10^5^ CFUs of UV-killed TIGR4 *S. pnuemoniae*. (**A**) Proportions and (**B**) numbers of granulocytes and agranulocytes in 4 and 48 h blister fluid samples as identified by forward and side scatter. (**C**) to (**E**) Analysis of the agranulocytes population using flow cytometry for CD3 surface expression.Proportion (**C**) an numbers (**D**) of CD3 negative agranulocytes. Proportions (**E**) and numbers (**F**) of CD3 positive agranulocytes. (**A**), (**C**), and (**E**) show representative flow cytometry plots from blood and blister fluid samples taken from 1 donor. Graph shown in (**A**) shows percentages of granulocytes and agranulocytes for 4 donor, analysed using one-way repeated measures ANOVA (*p < 0.5, **p < 0.01). Graphs shown in (**B**), (**D**), and (**F**) show data from 6 donors paired for the 4 h and 48 h results (lines join results from a single individual), and analysed using Wilcoxon matched-pairs signed rank test (*p < 0.5).

To analyse inflammatory cell recruitment at the site of intradermal *S. pneumoniae* challenge a blister was raised at the site of injection using a suction chamber at 4 h and 48 h post-injection (Fig. 1D). Fluid was aspirated from the blister, and its cellular content analysed using flow cytometry. Cells were gated for granulocytes, agranulocytes, CD3^−^ agranulocytes, and CD3^+^ T cells, which were further separated into CD8^+^, CD4^+^ and CD4^+^CD25^high^CD127^low^ subsets (Supplementary Fig. 1). The full gating strategy was validated using blood samples (Fig. 2A,C,E left hand panels). Initially granulocytes were the majority cell type present representing 77.7% ± 11.4% of all cells at 4 h post-injection but their absolute numbers decreased by 48 h (4 h 1.24 × 10^5^ ± 2.08 × 10^5^ cells per ml, 48 h 1.48 × 10^4^ ± 1.58 × 10^4^ cells per ml), and the proportion of granulocytes had decreased to 17.6% ± 14.6% of total cells by 48 h (Fig. 2A,B). Although the data were variable between volunteers, overall the cell numbers for the CD3^−^ agranulocyte population increased from 1.31 × 10^4^ ± 1.11 × 10^4^ cells per ml at 4 h to 4.52 × 10^4^ ± 2.79 × 10^4^ cells per ml at 48 h, suggesting increased monocyte recruitment over 48 h (Fig. 2C,D). CD3^−^ agranulocytes were 44.1% ± 33.2% of all cells at 4 h and 65.3% ± 25.0% of all cells at 48 h. The numbers of CD3^+^ T cells increased by 48 h compared to the 4 h blister fluid samples (4 h 1.68 × 10^4^ ± 1.98 × 10^4^ cells per ml, 48 h 8.40 × 10^4^ ± 6.92 × 10^4^ cells per ml) (Fig. 2E,F), suggesting significant recruitment of lymphocytes in response to intradermal *S. pneumoniae* challenge. The proportion of all cells that expressed CD3 increased from 15.9% ± 15.8% at 4 h to 55.6% ± 28.6% at 48 h (Table 1).

**Table 1:** Mean cell counts (per ml) and proportions of blister fluid cell subpopulations 4 and 48 h after intradermal inoculation of 7.5 × 10^5^ UV-killed TIGR4 *S. pneumoniae* bacteria (6 donors).

Cell type		Numbers per ml and proportions for each cell type			
		4 h		48 h	
		Mean	SD	Mean	SD
Granulocytes	Cell count	1.24 × 10^5^	2.08 × 10^5^	1.48 × 10^4^	1.58 × 10^4^
	% all cells	77.7	11.4	17.6*	14.6
CD3^−^ agranulocytes	Cell count	1.31 × 10^4^	1.11 × 10^4^	4.52 × 10^4^	2.79 × 10^4^
	% all cells	44.1	33.2	65.3	25.0
CD3^+^ T cells	Cell count	1.68 × 10^4^	1.98 × 10^4^	8.40 × 10^4^	6.92 × 10^4^
	% all cells	15.9	15.8	55.6*	28.6
CD8^+^ T cells	Cell count	6.46 × 10^3^	6.63 × 10^3^	3.22 × 10^4^	2.83 × 10^4^
	% all T cells	34.7	13.6	40.1	15.0
CD4^+^ T cells	cell count	7.65 × 10^3^	1.32 × 10^4^	3.48 × 10^4^	3.07 × 10^4^
	% all T cells	55.3	12.2	41.0	13.3
CD4-CD8-T cells	cell count	7.9 × 10^2^	8.94 × 10^2^	1.88 × 10^4^	3.00 × 10^4^
	% all T cells	6.7	7.7	17.4	15.2
CD4 + CD25^high^CD127^low^	cell count	8.79 × 10^3^	9.97 × 10^3^	1.27 × 10^4^	1.10 × 10^4^
	% all CD4 cells	21.7	13.6	39.0*	9.9

*P value versus 4 h =  < 0.05, paired T test.

### Changes in lymphocyte subsets numbers over time

**Figure 3:**
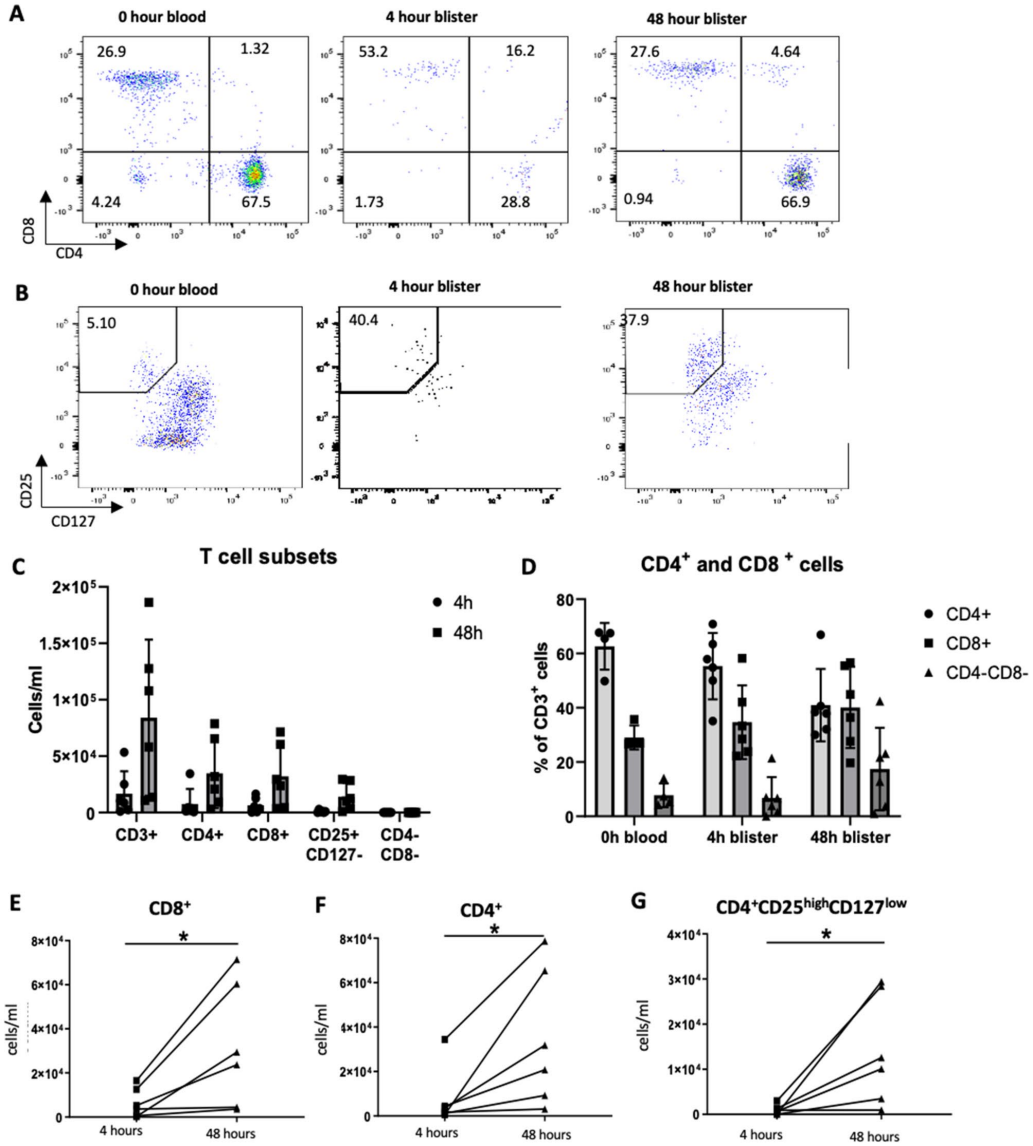
Lymphocyte subpopulations 4 and 48 h post-intradermal injection of killed *S. pnuemoniae* in the forearms of healthy volunteers. Data were obtained using flow cytometry to measure expression of lymphocyte cell surface markers in blister fluid cells obtained 4 and 48 h after intradermalin inoculation of 7.5 × 10^5^ CFUs of UV-killed TIGR4 *S. pnuemoniae*. (**A**) to (**B**) representative flow cytometry plots of CD4/CD8 (**A**) and CD25^+^CD127 (**B**) populations in blood and blister fluid samples taken from 1 donar. (**C**) Total cell number of CD3^+^, CD4^+^, CD8^+^, CD25^+^CD27^−^, and CD4^−^CD8^−^ cells in 4 (left column each cell type, circle symbols) and 48 (right column each cell type , square symbols) hour blister fluid samples. (**D**) Proprtion of CD3^+^ cells that were CD4^+^ (circle symbols), CD8^+^ (square sumbols), CD4^−^CD8^−^ (triangle symbols) in pre-infection blood samples, and 4 and 48 h blister fluid. For (**C**) and (**D**) each symbols represents data from an individuals volunteers, columns represent means, and error bars SDs. (**E**), (**F**), and (**G**) cell numbers per ml of blister fluid for CD4^+^ (**E**), CD8^+^ (**F**), and CD25^+^CD27^−^ (**G**) subsets for 6 donors paired for the 4 h anf 48 h results (lines join results from a single individual), and analysed using Wilcoxon matched-pairs signed rank test (*p < 0.5).

Within the CD3^+^ T cell population, there were increases in recruited cell numbers for both CD4^+^ and CD8^+^ cells (CD4^+^ 4 h 7.65 × 10^3^ ± 1.32 × 10^4^, 48 h 3.48 × 10^4^ ± 3.07 × 10^4^; CD8^+^ 4 h 6.46 × 10^3^ ± 6.63 × 10^3^, 48 h 3.22 × 10^4^ ± 2.83 × 10^4^) (Fig. 3A,C,D,E,F). Similar to the data for blood, CD4 cells were the predominant lymphocyte population in blister fluid at 4 h (CD4^+^ 55.3% ± 12.2%, CD8^+^ 34.7 ± 13.6%). However, by 48 h the blister fluid contained similar proportions of CD4^+^ and CD8^+^ T cells (CD4^+^ 41.0% ± 13.3%, CD8^+^ 40.1% ± 15%) (Fig. 3D). In addition, the cell numbers for the CD4^+^CD25^high^CD127^low^ population increased between 4 to 48 h (4 h 8.79 × 10^3^ ± 9.97 × 10^3^, 48 h 1.27 × 10^4^ ± 1.10 × 10^4^) (Fig. 3B,C,G), indicating that significant numbers of Treg cells were recruited to the site of *S. pneumoniae* challenge. The CD4^+^CD25^high^CD127^low^ population represented 21.6% ± 13.6% of all CD4^+^ T cells at 4 h, increasing to 39.0% ± 9.9% by 48 h (Table 1, *P* = 0.047 paired *T* test). Overall, the blister fluid cellular analysis using flow cytometry suggested that although granulocytes (likely neutrophils) predominated in the 4 h blister fluid, by 48 h post-*S. pneumoniae* challenge CD3^−^ agranulocytes (likely monocytes/macrophages) and CD3^+^ T cells (which included a high proportion of putative CD4^+^CD25^high^CD127^low^ Treg cells) were present in high numbers and dominated the cellular inflammatory response. Recruited cell subset numbers and relative proportions are summarised in Table 1.

### Recruited CD4^+^ T cells were predominantly effector/memory CD45RA^−^ and express the tissue-homing receptor CCR6

**Figure 4:**
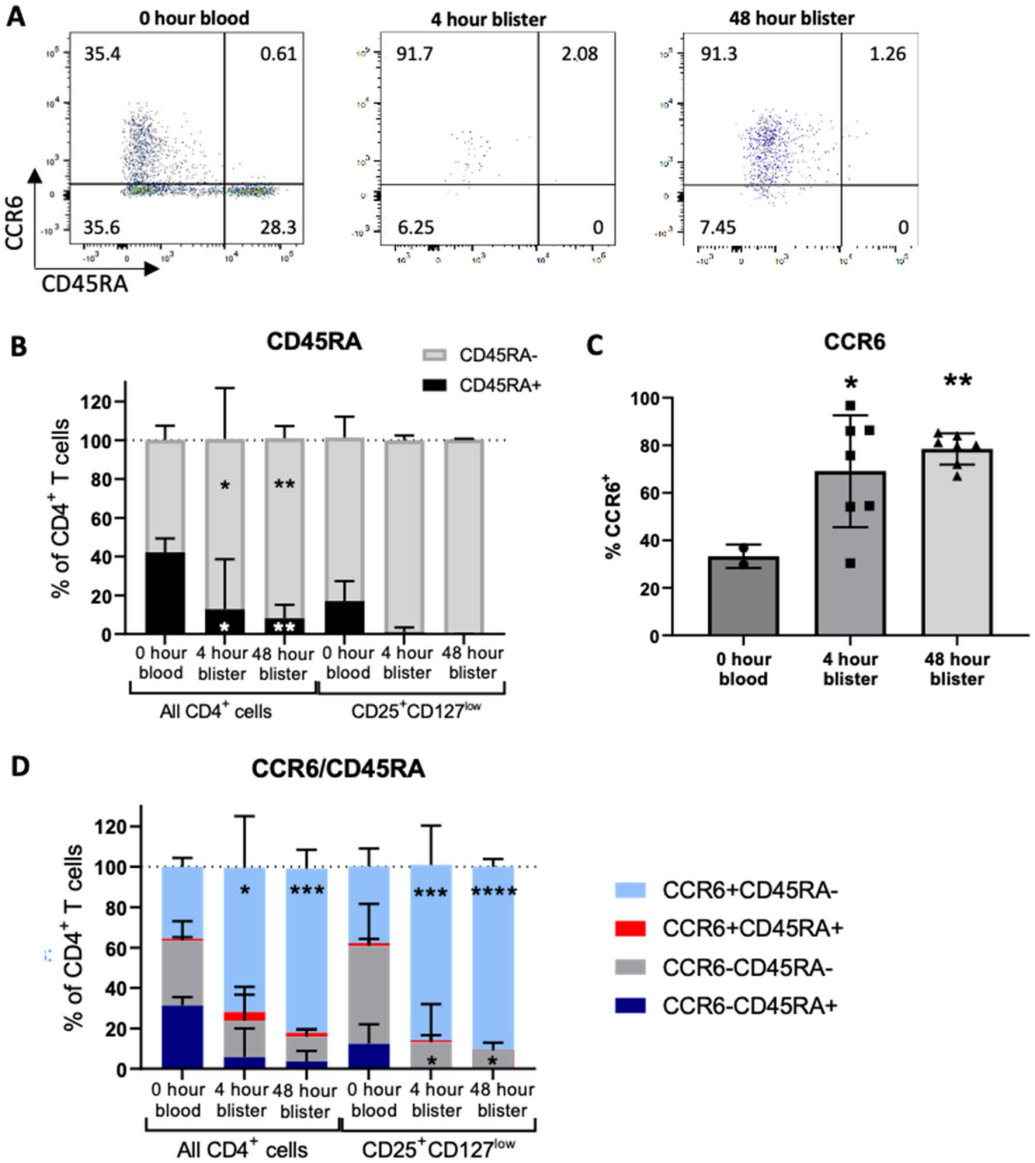
CD4^+^ T cells CD45RA and CCR6 expression at the site of intradermal *S. pnuemoniae* challenge. Data were obtained by flow cytometry of blister fluid collected at 4 h and 48 h after intradermal inoculation 7.5 × 10^5^ CFUs of UV-killed TIGR4 *S. pnuemoniae* (6 donors) and in pre-challenge peripheral blood (2 to 4 donors). (**A**) reprentative flow cytometry plots of CCR6 and CD45RA expression. (**B**) Mean (SD) percentages of CD45RA^−^ (grey) and CD45RA^+^ (black) cells in the CD4^+^CD25^+^CD127^low^ populations analysed by two-way ANOVA and Tukey’s multiple comparisons test comparing 0 h blood samples with the 4 h and 48 h blister samples (*p < 0.05; **p < 0.01). (**C**) Mean (SD) percentages of CD4^+^ T cells expressing CCR6. Data were analysed using one-way ANOVA (*p* < 0.01) with Dunnett’s multiple comparisons test (*p < 0.05; **p < 0.01). (**D**) Mean (SD) expression of both CD45RA and CCR6 by the CD4^+^ T cell and CD4^+^CD25^+^CD127^low^ populations analysed by two-way ANOVA and Tukey’s multiple comparisons test comparing CCR6^+/−^CD45RA^+/−^ expression in the 0 h blood samples wit the 4 h and 48 h blister samples (*p < 0.05; ***p < 0.001; ****p < 0.0001).

Expression of CD45RA was used to further characterise blister fluid CD4^+^ T cells into naive (CD45RA^+^) or effector/memory (CD45RA^−^) T cell populations^27^**.** In pre-challenge peripheral blood samples, the CD4^+^ T cell population both contained CD45RA^+^ cells (42% ± 7%) and CD45RA^−^ cells (58.1% ± 7%) (Fig. 4A,B). In blister fluid the proportions of CD45RA^−^ CD4^+^ T cells were higher compared to peripheral blood (CD45RA^−^ CD4^−^ T cells in 0 h blood 58% ± 7%, at 4 h 87% ± 26%, and 48 h 93% ± 6%) (Fig. 4A,B). An increase in the proportion of CD45RA^−^ cells also occurred with the putative CD4^+^CD25^high^CD127^low^ Treg cell population, with virtually all these cells being CD45RA^−^ in 4 and 48 h blister fluid samples (4 h 99% ± 2%, 48 h 100% ± 1%, versus 84% ± 11% for peripheral blood) although this increase was not statistically significant (Fig. 4A,B). These results indicated that most of the blister fluid CD4 cell population and almost all of the Treg cells had had antigen experience and an effector/memory phenotype.

Expression of the tissue-homing receptor CCR6 by CD4^+^ T cells is restricted to Treg, Th17, Th22, and Th1 subsets of Th17 origin^28–32^. Hence, CCR6 expression was measured to further subclassify the CD4^+^ T cell populations present in the blister fluid. A higher proportion of blister fluid CD4^+^ T cells were CCR6^+^ than in pre-challenge blood (peripheral blood 36% ± 5%; 4 h blister 76% ± 20%, 48 h 83% ± 9%) (Fig. 4C). These data suggested that CD4^+^ T cell recruitment to blister fluid was dominated by tissue-homing/resident Treg, Th22, Th17 or non-classical Th1 cell subsets. To further characterise the functional status of the CD4 subsets, co-expression of CCR6 and CD45RA was assessed. The dominant fraction of CD4^+^ T cells in 4 h and 48 h blister fluid samples were CCR6^+^CD45RA^−^ (72% ± 25% at 4 h, and 81% ± 9% at 48 h, compared to 36% ± 5% for pre-challenge peripheral blood) (Fig. 4C,D). Smaller proportions of blister fluid CD4^+^ T cells were CD45RA^−^CCR6^−^ (18% ± 17% at 4 h and 12% ± 4% at 48 h), a cell subset that may contain classical Th1/Th2 memory/effector T cell subsets. The proportions of blister fluid CD4^+^ T cells in the CD45RA^+^ groups ± CCR6 were small and highly variable owing to the small numbers of cells present in these groups (CCR6^−^CD45RA^+^ 6% ± 14% at 4 h, 4% ± 5% at 48 h, CCR6^+^CD45RA^+^ 4% ± 9% at 4 h, 2% ± 2% at 48 h). Collectively, the majority of the CD4^+^ T cells in the blister fluid expressed CCR6 without CD45RA, suggesting they were effector/memory cells predominantly from the Treg, Th22, and/or Th17 origin subsets.

### Blister fluid cytokine levels suggest a Th17 response to* S. pneumoniae* challenge

**Figure 5:**
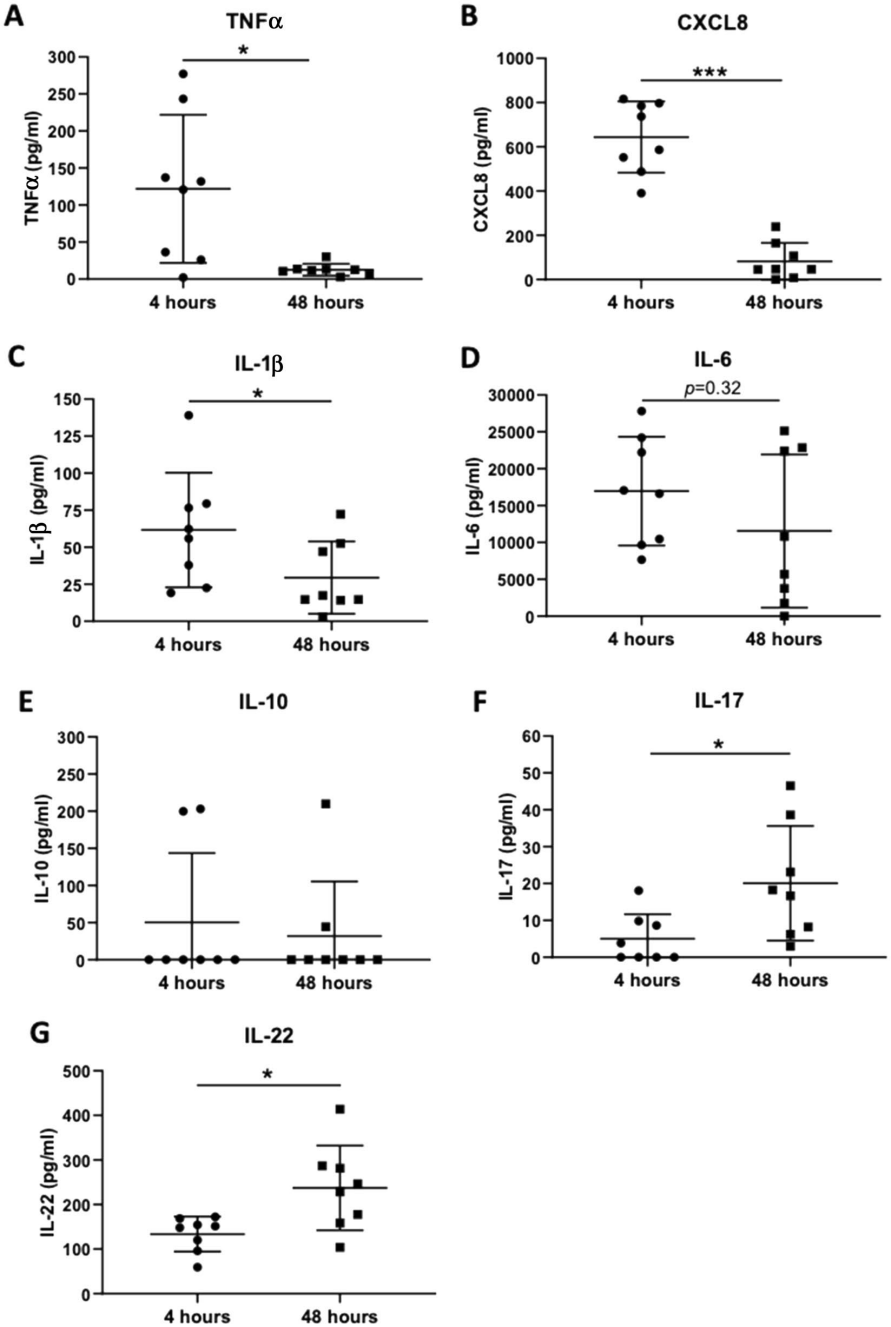
Cytokine concentrations in the blister fluid at the site of *S. pnuemoniae* intraderam; injection. (**A**) TNFα, (**B**) CXCL8, (**C**) IL-1β, (**D**) IL-16, (**E**) IL-10, (**F**) IL-17, and (**G**) IL-22 concentration (pg per ml) measured by ELISA in blister fluid supernatants collected 4 and 48 h after intradermal injection of 7 × 10^5^ CFUs of UV-killed TIGRS *S. pnuemoniae* for 8 donors. Each symbols represents data from one donar, bars represent mean values and error bars SDs. The reults were analysed using Mann Whitney U tests (*p < 0.05; ***p < 0.0001).

The concentrations of TNFα, CXCL8, IL-1β, IL-6, IL-17, IL-22 and IL-10 present in the blister fluid 4 h and 48 h post-*S. pneumoniae* challenge were determined by ELISA. Levels of TNFα, CXCL8 and IL-1β were raised at 4 h and showed statistically significant decreases by 48 h (Fig. 5A–C), corresponding with the observed decrease in granulocytes over time (Fig. 2A). IL-6 levels were markedly raised at both 4 and 48 h (Fig. 5D), but significant levels of IL-10 were only detected in a minority of subjects (Fig. 5E). Levels of IL-17 and IL-22 increased in blister fluid between 4 and 48 h (Fig. 5F,G), corresponding to the recruitment of CCR6^+^ CD4^+^ T cells by 48 h (Figs. 3, 4) and compatible with an accumulation of Treg, Th22, Th17 and Th17-derived T cell subtypes.

## Discussion

Accurately defining human immune and inflammatory responses to challenge with *S. pneumoniae* is essential for our understanding of disease pathogenesis. An effective immune and inflammatory response is necessary for bacterial clearance, but can also have negative consequences for the host causing consolidation of the lung during pneumonia, neuronal damage in meningitis, and severe complications of infection such as septic shock or acute respiratory distress syndrome (ARDS)^33,34^. We have described a new model of intradermal injection of killed *S. pneumoniae* human challenge and demonstrated that the model can define the inflammatory and immunological responses at the site of infection.

Using the model, we have shown that after intradermal injection of killed *S. pneumoniae* there is a large increase in local blood flow to the site of injection, an initial influx of granulocytes (likely neutrophils), followed by a more delayed influx of monocytes and lymphocytes. The early 4 h cytokine/chemokine response was dominated by TNF, IL6 and CXCL8; of these only the IL6 response persisted at 48 h. This pattern of the initial innate response to *S. pneumoniae* is very similar to that described using mouse models of infection^13,17,18^, and compatible with the data obtained using samples from human infection which shows a marked neutrophilic infiltrate into the lungs during pneumonia associated with high levels of inflammatory cytokines^35,36^. The marked dynamic changes over time in white cell populations and cytokines demonstrate the blister fluid data reflects a strong dermal inflammatory response to injection of killed *S. pneumoniae*. For comparison, previously published data show the response to intradermal injection of PBS alone resulted in very low blister fluid cytokine levels (eg under 10 pg/ml for TNFα, IL-1β, and IL-6), and neutrophil and T cell numbers that were approximately 2 log_10_ and 1 log_10_ lower respectively than seen in response to *S. pneumoniae*^37^. Our data show that the intradermal challenge model can be used to assess cellular recruitment and cytokine responses to *S. pneumoniae* at specified timepoints, and could be used to investigate how these responses are affected by host factors such as treatment with immunomodulators, age, or disease. However, future studies should include PBS treated data as controls to ensure differences between different subjects reflects the response to *S. pneumoniae* rather than to the injection and blister formation. Responses could also be compared between different *S. pneumoniae* strains including genetically modified strains missing specific virulence factors or pro-inflammatory pathogen associated molecular patterns (eg lipoproteins)^38^. The model is based on a previously described model using intradermal injection of killed *Escherichia coli* that described innate immune defects in patients with inflammatory bowel disease^39–41^, demonstrating the potential for the model to identify clinically significant differences between patient groups. As the model recovers significant numbers of immune and inflammatory cells from the site of infection, it has the potential to allow a very detailed analysis of immune responses to *S. pneumoniae* using single cell RNAseq or isolation of antigen specific lymphocytes for further molecular characterisation.

Interpretation of data obtained using the intradermal model needs to take into account that for safety reasons the model uses killed bacteria. Hence, the model will not identify specific effects of bacterial/host interactions that depend on live bacteria such as active cell invasion and modulation of host signalling pathways. The model is limited to investigating the response to a single short lived exposure to *S. pneumoniae*, with each subject having two samples in total taken (e.g. 4 and 24 h, or 4 and 48 h). The significant variability in results between subjects and the low numbers of cells obtained from the blister fluid will make it hard to identify smaller differences between groups of subjects (eg due to age or sex) or time points, and provides technical limits on the assays that can be easily performed. Significantly larger numbers of subjects are likely to be needed to identiy biological differences between patient groups. The skin is not a natural site of *S. pneumoniae* infection and although the data will likely reflect what happens at inaccessible infection sites such as the lung and brain, there are also likely to be some differences. However, skin infection has been used successfully to characterise systemic alterations in human responses to infection^40^, and our data indicate that immunological responses to *S. pneumoniae* in the skin resemble those previously described for the lung. For example, as discussed above the innate responses seen after intradermal challenge with killed *S. pneumoniae* were very similar to those seen in mouse lung after live *S. pneumoniae* infection. Furthermore, in contrast to blood CD4^+^ T cells very few lung resident CD4^+^ T cells are CD45RA^+^^42^, and this resembles the CD4^+^ T cell response after intradermal injection of *S. pneumonia*e shown here. Combining data from the established live pneumococcal nasopharyngeal colonisation model and from acute studies in humans with natural infection^35^ with the detailed immunological data obtained using the intradermal model will provide a comprehensive overview of human innate and adaptive responses to *S. pneumoniae*. Animal studies directly comparing immune responses to live and dead *S. pneumoniae* in mice with adaptive immune responses to *S. pneumoniae* from previous exposure would be helpful in interpreting the effects of using killed bacteria in the intradermal human challenge model.

We have used the intradermal challenge model to define the lymphocyte response to *S. pneumoniae* challenge in humans. The results show that 4 h post-challenge there are only very low levels of lymphocytes in blister fluid, but by 48 h lymphocytes are the dominant cell population. This influx of lymphocytes was dominated by T cells rather than B cells. There was a slightly greater relative increase in CD8^+^ T cells compared to CD4^+^ T cells with these two subsets each contributing about 40% of the recruited lymphocyte population at 48 h. The functional relevance of this high level of recruited CD8^+^ T cells for immunity to an extracellular bacterial pathogen is not clear. Previous investigation^43^ using a mouse model of *S. pneumoniae* infection in immune naïve animals demonstrated an unexpectedly important protective role for CD8^+^ T cells; the mechanism(s) involved remain unclear. The increase in blister fluid levels of IL17 and IL22 and the high proportion (83%) of CD4^+^ T cells that were CCR6^+^ at 48 h suggest that recruited CD4^+^ T cells included a significant population of Th17 cells. Corresponding with these findings, mouse models have shown important roles for enhanced Th17 responses to reinfection including from CD4^+^ T resident memory T cells to naturally acquired protection against re-infection with *S. pneumoniae*^44,45^. The CD4^+^ T population also consisted of a surprisingly high number of likely Treg cells (CD4^+^CD25^high^CD127^low^, 39% of all CD4^+^ T cells at 48 h). Most of these potential Tregs cells were CCR6 positive and CD45RA negative suggesting they are memory cells recruited in response to *S. pneumoniae*, but their functional role is unclear. There are only very limited data on the role of Tregs during *S. pneumoniae* infection; mouse data suggest Tregs are protective, modulating the duration of *S. pneumoniae* nasopharyngeal colonisation and preventing the development of septicaemia during pneumonia, perhaps by preventing epithelial/endothelial barrier breakdown^6,19^. However, these data were obtained using immune naïve mice and also run counter to data showing Tregs usually increase susceptibility to infection with other pathogens by suppressing inflammatory responses^46,47^. The recruitment of Tregs to the site of *S. pneumoniae* infection may be important for resolution of the inflammatory responses. The intradermal model provides a method for defining this potential role of Tregs during *S. pneumoniae* infection using human tissue and could be important for our understanding of how pneumonic consolidation usually resolves without causing persistent lung damage. Confirmation of the functional identify of the different CD4^+^ T cell populations recruited to the blister fluid at the site of *S. pneumoniae* intradermal challenge would require additional assays such as intracellular cytokine staining (Th17 CD4^+^ T cells) or suppression of inflammatory responses (CD4^+^CD25^high^CD127^low^ as Tregs), but any additional analyses to flow cytometry were prevented by the low numbers of recovered cells. More detailed characterisation of the CD4^+^ T and CD8^+^ T cell sub-populations, their influence on the immunological and inflammatory response to *S. pneumoniae*, and the identification of which antigens they recognise are all important subjects for future investigation using the intradermal challenge model.

In summary, we describe a novel safe human challenge model using intradermal injection of killed *S. pneumoniae* that results in an acute inflammatory response similar to that seen in animal models of pneumonia, and which was then followed by an influx of lymphocytes by 48 h which included surprisingly high proportions of CD8^+^ T cells and probable memory Tregs cells. This intradermal model can be used to characterise in detail the human immune response to *S. pneumoniae* challenge, and can therefore to investigate why age and a range of diseases and/or treatments that modulate the immune response are associated with increased susceptibility to *S. pneumoniae*. Furthermore, the model could be readily adapted for investigating multiple other bacterial pathogens, thereby extending the range of infections for which relevant human data can be obtained.

## Methods

### Healthy volunteers

A total of 11 (7 male, 4 female) healthy volunteers aged 18 to 35 years old were recruited for this study, with lymphocyte data obtained from a subset of 6 subjects (5 male, 1 female). None of the subjects had received vaccination against *S. pneumoniae*. The experiments were approved by the UCL Research Ethics Committee (ref. 7577/001) with written informed consent obtained from all participants, and all methods were performed in accordance with the relevant guidelines and regulations.

### Preparation of UV-killed S. pneumoniae

Capsular serotype 4 *S. pneumoniae* TIGR4 (a gift from Professor Jeffrey Weiser, University of Pennsylvania) was grown overnight on Columbia agar blood plate (SGL, 8022) at 37 °C in 5% CO_2_, then transferred into 15mls of autoclaved THY broth (Sigma Aldrich, T1438) supplemented with 0.5% yeast extract (Sigma Aldrich, Y1625) in a 50 ml falcon tube (Greiner, T2318-500EA) and cultured at 37 °C in 5% CO_2_ with the cap loosely replaced. On reaching an optical density_600_ of 0.4 (approximately 1 × 10^8^ colony forming units [CFU] per ml) the *S. pneumoniae* cultures were centrifuged at 13,000×*g* for 10 min, the supernatant discarded, and bacteria re-suspended in sterile PBS (Sigma Aldrich, D8537) in a sterile petri dish then exposed to UV light (302 nm, ChemiDoc; Bio-Rad, UK) for 1 h. The UV-killed *S. pneumoniae* were collected into a sterile 50 ml falcon tube, washed with sterile normal saline and centrifugation at 13,000×*g* for 10 min. Aliquots of 1.5 × 10^8^ UV-killed *S. pneumoniae* in 1 ml of sterile saline were frozen at −80 °C in autoclaved 10% glycerol (VWR 24,388.260) diluted in distilled water until required for injection. The *S. pneumoniae* in the UV stocks were confirmed to be dead by culture (probable limit of detection of < 1 in 1 million bacteria) by the University College London Hospitals Microbiology department.

### Intradermal injection of UV-killed S. pneumoniae and induction of suction blisters

The protocol for intradermal injection of killed *S. pneumoniae* was adapted from Motwani et al*.* 2016^39^. Aliquots of UV killed *S. pneumoniae* containing 7.5 × 10^5^ bacteria were resuspended in 100 μl of sterile saline and inoculated into the anterior aspect of the forearms of a healthy volunteer by intradermal injection. Dose finding experiments established that 7.5 × 10^5^ killed TIGR4 *S. pneumoniae* induced visible erythema in most subjects, whereas lower doses often resulted in no visible signs of the bacterial challenge. Each volunteer had one injection in each arm. At pre-specified time points, a suction chamber with a 10 mm diameter hole connected to a negative pressure instrument (NP-4, Electronic Diversities Ltd., MD, USA) was secured over the site of injection, and negative pressure gradually applied until a fully formed blister was visible. The pressure was then gradually returned to the baseline, and the blister pierced with a 23G needle (Fisher Scientific, NN-2332R) and the fluid collected using a 200 μl pipette. The blister area of the forearm of the volunteers was cleaned with 0.5% cetrimide spray (Savlon) and a large protective dressing applied (Mepore).

### Laser doppler imaging

At specified time points before blister induction doppler scans were taken of the intradermal injection site using a Laser Doppler Imager (Moor LDI-HIR, Moor Instruments Ltd, UK) and the colour-coded image analysed using moorLDI software (Version 5). Blood flow (referred to as arbitrary “perfusion units”) was quantified by multiplying the pixel number by the mean blood flow signal after subtraction of pixels below a fixed threshold of 300 perfusion units.

### Cell preparation and flow cytometry

After lysing red blood cells with ACK buffer (Lonza, 10-548E), leukocytes were isolated from bloodby an initial wash step in PBS with centrifugation at 400×*g* for 5 min, followed by resuspension in staining buffer (PBS supplemented with 0.5% fetal bovine serum [FBS, Thermo Fisher Scientific, 26140079] and 0.4% 2 mM EDTA [ThermoFisher Scientific, 15575020]). Leukocytes were isolated from blister fluid by centrifugation at 400×*g* for 5 min, removal of the supernatant for cytokine analysis, then re-suspendion in ACK lysis buffer for 1 min, followed by a PBS washing step, centrifugation at 400×*g* and re-suspension in 100 μl of staining buffer. Cell counts were obtained using a haemocytometer and Trypan blue to exclude dead cells. For flow cytometry, 10^6^ blood/blister cells were used in 100 μl of staining buffer containing 1:1000 dilution of LIVE/DEAD Fixable Blue Dead Cell Stain (Invitrogen, L23105) and when appropriate the following antibodies: 2.5 μl CD3 BUV395 (BD Biosciences, 563548), 2.5 μl CD8 PerCP-Cy5.5 (BD Biosciences, 560662), 10 μl CD4 APC (BD Biosciences, 555349), 10 μl CD25 PE (BD Biosciences, 555432), 2.5 μl CD127 BV421 (BD Biosciences, 562437), 2.5 μl CD45RA BV605 (BD Biosciences, 562886), 2.5 μl CCR6 BB515 (BD Biosciences, 564479). After incubation at 4 °C for 25 min in the dark, the samples were made up to 1 ml with staining buffer, centrifuged at 400×*g* for 5 min, then resuspended in 300 μl of staining buffer containing 2% paraformaldehyde. The LSR II (BD Biosciences) flow cytometer and FlowJo software (Version 10) was used to analyse the cells, removing cell debris by exclusion of very low forward and side scatter area (FSC-A and SSC-A) data, doublets by excluding outliers on the forward scatter height (FSC-H) and FSC-A profile, and dead cells by exclusion of LIVE/DEAD stain positive cells. Compensation controls were prepared using 1 μl of antibody and 1 drop of OneComp eBeads (eBioscience, 01-1111-41). FACSDiva software was used to calculate the compensation needed for the antibody panel combinations used.

### Enzyme-linked immunosorbent assay (ELISA)

Cytokine concentrations were measured in the blister fluid using TNFα (DY210), IL-6 (DY206), IL-1β (DY201), IL-10 (DY217B), IL-17 (DY317), IL-22 (DY782), IL-8 (DY208) kits from R&D Systems as per the manufacturer’s instructions and streptavidin-HRP and tetramethylbenzidine (Invitrogen, 002023) detection as previously described^38^. Absorbance was read on a plate reader (Versamax, Sunnyvale, CA, USA) at 450 nm minus 540 nm. Concentration of cytokines in the samples were calculated by comparison against the standard curve.

### Statistical analyses

Statistical analyses were performed using GraphPad Prism Version 8 software. Where two groups were compared, Mann–Whitney test was used for unpaired data or Wilcoxon matched-pairs signed rank test for paired data. Where more than two groups were being compared, one-way analysis of variance (ANOVA) or Kruskal–Wallis test (for non-parametric data) were used. For paired data with more than two groups, the repeated-measures one-way ANOVA was used. Post-tests (Dunnett’s multiple comparison test, Dunn’s multiple comparisons test or Tukey’s multiple comparisons test) were conducted on analyses with more than two groups. Two-tailed analyses were used in all statistical tests. Unless otherwise stated, error bars show standard deviation (SD) from the mean. P values of < 0.05 were considered significant.

## Supplementary Information

Supplementary Information.

## Data Availability

The authors declare that the data supporting the findings of this study are available within the paper and its supplementary information files, or in the source data file.
