# Author Correction: A novel *Streptococcus pneumoniae* human challenge model demonstrates Treg lymphocyte recruitment to the infection site

**DOI:** 10.1038/s41598-022-10026-0

**Published:** 2022-04-05

**Authors:** Gabriella Szylar, Riccardo Wysoczanski, Helina Marshall, Daniel J. B. Marks, Ricardo José, Michael R. Ehrenstein, Jeremy S. Brown

**Affiliations:** 1 Centre for Inflammation and Tissue Repair, UCL Respiratory, Division of Medicine, Rayne Building, 5 University Street, London, WC1E 6JF UK; 2 Centre for Rheumatology, UCL Division of Medicine, Rayne Building, 5 University Street, London, WC1E 6JF UK; 3 Centre for Molecular Medicine, UCL Division of Medicine, Rayne Institute, 5 University Street, London, WC1E 6JF UK

Correction to: *Scientific Reports* https://doi.org/10.1038/s41598-022-07914-w, published online 07 March 2022

The original version of this Article contained an error in the spelling of the author Michael R. Ehrenstein which was incorrectly given as Michael E. Ehrenstein. The original Article has been corrected.

