# Supplementary figures and images for: A novel Streptococcus pneumoniae human challenge model demonstrates Treg lymphocyte recruitment to the infection site
Source: Sci Rep. 2022 Mar 7;12:3990. doi: 10.1038/s41598-022-07914-w (PMC8901783; doi:10.1038/s41598-022-07914-w)

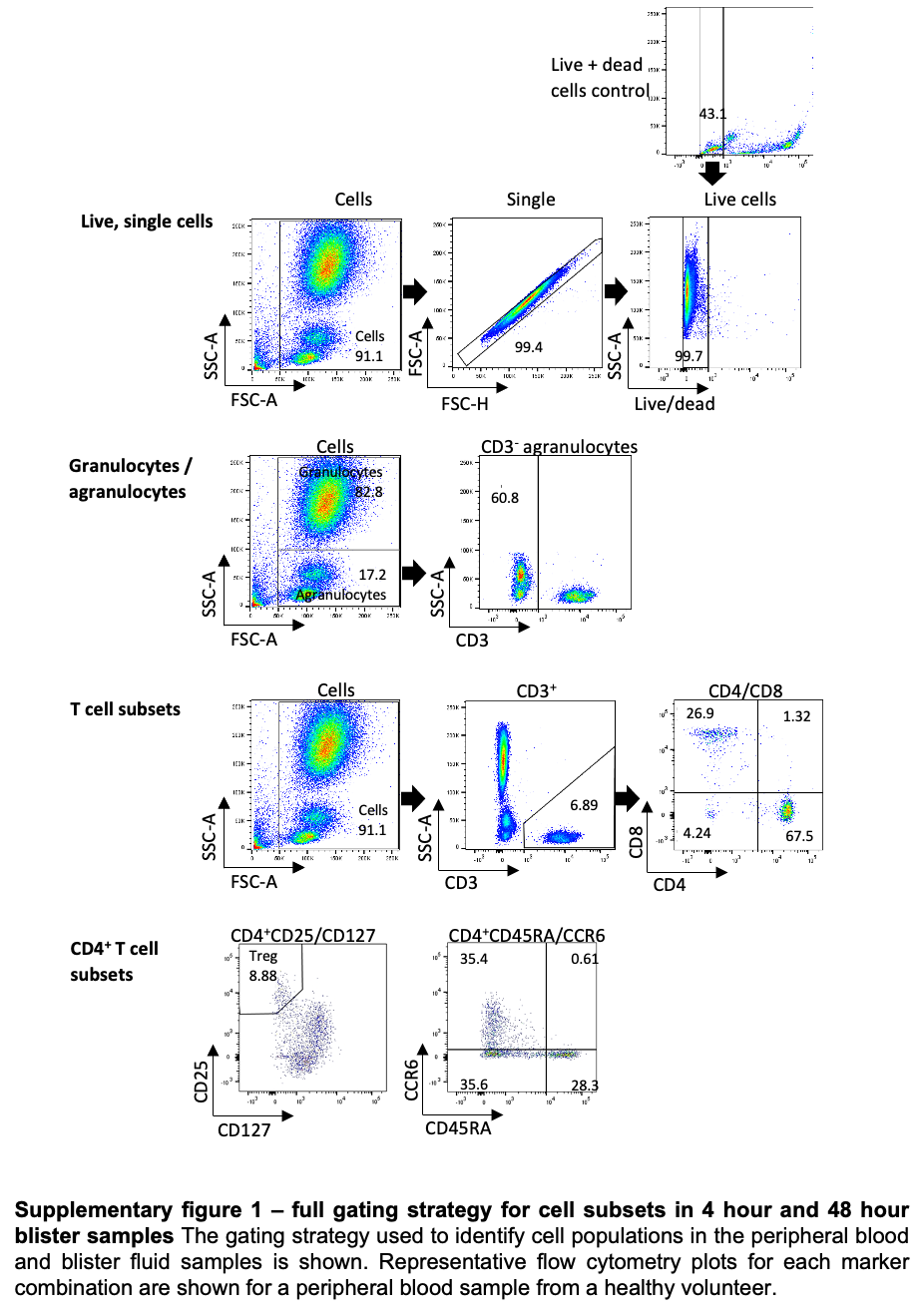

Supplement: Supplementary file 1 — Supplementary Information. [file 41598_2022_7914_MOESM1_ESM.docx]
